# Transcriptional Regulation of *Autophagy-Related Genes* by Sin3 Negatively Modulates Autophagy in Magnaporthe oryzae

**DOI:** 10.1128/spectrum.00171-23

**Published:** 2023-05-16

**Authors:** Zhongling Wu, Huanbin Shi, Yuan Li, Fei Yan, Ziyue Sun, Chuyu Lin, Mengting Xu, Fucheng Lin, Yanjun Kou, Zeng Tao

**Affiliations:** a State Key Laboratory for Managing Biotic and Chemical Threats to the Quality and Safety of Agro-products, Institute of Biotechnology, Zhejiang University, Hangzhou, China; b Ministry of Agriculture Key Laboratory of Molecular Biology of Crop Pathogens and Insects, Institute of Biotechnology, Zhejiang University, Hangzhou, China; c State Key Lab of Rice Biology and Breeding, China National Rice Research Institute, Hangzhou, China; d Hainan Institute, Zhejiang University, Sanya, China; South China Agricultural University Integrative Microbiology Research Centre

**Keywords:** histone deacetylation, rice blast, fungal pathogen, transcriptional regulation, *ATG8*

## Abstract

Autophagy is a conserved degradation and recycling pathway in eukaryotes and is important for their normal growth and development. An appropriate status of autophagy is crucial for organisms which is tightly regulated both temporally and continuously. Transcriptional regulation of *autophagy-related genes* (*ATGs*) is an important layer in autophagy regulation. However, the transcriptional regulators and their mechanisms are still unclear, especially in fungal pathogens. Here, we identified Sin3, a component of the histone deacetylase complex, as a transcriptional repressor of *ATGs* and negative regulator of autophagy induction in the rice fungal pathogen Magnaporthe oryzae. A loss of *SIN3* resulted in upregulated expression of *ATGs* and promoted autophagy with an increased number of autophagosomes under normal growth conditions. Furthermore, we found that Sin3 negatively regulated the transcription of *ATG1*, *ATG13*, and *ATG17* through direct occupancy and changed levels of histone acetylation. Under nutrient-deficient conditions, the transcription of *SIN3* was downregulated, and the reduced occupancy of Sin3 from those *ATGs* resulted in histone hyperacetylation and activated their transcription and in turn promoted autophagy. Thus, our study uncovers a new mechanism of Sin3 in modulating autophagy through transcriptional regulation.

**IMPORTANCE** Autophagy is an evolutionarily conserved metabolic process and is required for the growth and pathogenicity of phytopathogenic fungi. The transcriptional regulators and precise mechanisms of regulating autophagy, as well as whether the induction or repression of *ATGs* is associated with autophagy level, are still poorly understood in M. oryzae. In this study, we revealed that Sin3 acts as a transcriptional repressor of *ATGs* to negatively regulate autophagy level in M. oryzae. Under the nutrient-rich conditions, Sin3 inhibits autophagy with a basal level through directly repressing the transcription of *ATG1-ATG13-ATG17*. Upon nutrient-deficient treatment, the transcriptional level of *SIN3* would decrease and dissociation of Sin3 from those *ATG*s associates with histone hyperacetylation and activates their transcriptional expression and in turn contributes to autophagy induction. Our findings are important as we uncover a new mechanism of Sin3 for the first time to negatively modulate autophagy at the transcriptional level in M. oryzae.

## INTRODUCTION

Autophagy is an evolutionarily conserved metabolic process that maintains intracellular energy balance in eukaryotes and plays important roles in development, cellular differentiation, and adaption to environmental stresses (1–3). The induction of autophagy is usually associated with autophagosome biogenesis, which is a double-layer membrane structure that wraps up some damaged proteins or organelles and transports them to lysosomes or vacuoles for degradation and circulation, thereby maintaining energy homeostasis in eukaryotes (1, 4). An appropriate state of autophagy is beneficial to organisms, and either increased or decreased autophagy can be detrimental (1, 5). Thus, the process of autophagy must be regulated precisely. The regulation of autophagy is affected by a series of *autophagy-related genes* (*ATGs*) and intracellular pathways (2). The expression of many *ATGs* and the levels of their encoding proteins are substantially increased under autophagy-inductive conditions, which implied that such an induction would be critical for the optimal maintenance of autophagy efficiency and energy homeostasis (6–8). It has long been thought that the regulation of autophagy is a posttranslational process; however, recent studies have indicated that transcriptional regulators and histone modifications are also involved in the transcriptional regulation of *ATGs* (7, 9, 10).

In eukaryotes, the repeating subunit of chromatin is the nucleosome, formed by 147-bp DNA wrapped around a histone octamer containing two copies of each of the following histones: H2A, H2B, H3, and H4 (10). The chromatin state of the target locus affects the accessibility of transcriptional machinery and is closely associated with their transcriptional expression (10). Histone modifications and transcriptional factors have been identified to be involved in the transcriptional regulation of autophagy negatively or positively in model organisms, such as yeast and mammals (8, 11). In yeast, the levels of acetylation of lysine 56 on histone H3 (H3K56ac) and H4K16ac are changed globally after starvation induction in yeast (6, 7, 12). Histone acetyltransferase KAT8 and histone deacetylase SIRT1 coregulate the transcriptional expression of *ATGs* which associates with dynamic enrichment of H4K16ac (7, 13). Histone demethylase *Rph1* is a conserved transcriptional repressor of *ATGs* and maintains autophagy at a basal level under normal conditions (14). The inhibition of histone H2B monoubiquitination and deletion of the genes encoding core subunits of polymerase-associated factor 1 complex (Paf1C) induce autophagy activation with increasing *ATG* expression (15, 16). Histone arginine methyltransferase 1 contributing to histone H3R17 dimethylation was identified as a transcriptional coactivator of autophagy-related and lysosomal genes (8). In addition, transcriptional regulatory gene *Pho23* and zinc finger protein *ZKSCAN3* have been reported as master transcriptional repressors for autophagy through directly targeting *ATGs* (17–19). Transcriptional factors *FoxO3* and *TFEB* have been shown to activate the expression of autophagy genes in response to various stresses (20, 21). However, the precise mechanism in the regulation of autophagy at the transcriptional level is still unclear, especially in plant-pathogenic fungi.

Accumulating evidence indicates that autophagy is required for the growth and pathogenicity of phytopathogenic fungi (3, 5, 22–24). During the infection process of pathogens, autophagy is required for the formation and development of the infectious structure, thereby affecting the virulence of the pathogen (3, 5). The rice blast fungus, Magnaporthe oryzae, seriously threatens the production of rice worldwide and results in a 10 to 30% reduction in yield every year (25). In M. oryzae, conidial autophagy is critical for the formation of functional appressoria, and numerous *ATGs*, such as *ATG1*, *ATG5*, *ATG8*, *ATG12*, *ATG14-ATG16*, and *ATG18*, were identified to be required for fungal development and pathogenicity (3, 26–29). While the majority of studies addressing the induction of autophagy have focused upon the cytoplasmic regulation of ATG proteins in M. oryzae, the knowledge regarding nuclear events, such as the transcriptional regulation of *ATGs*, is still unexplored (3). Recently, *MoSNT2* was reported to be involved in the transcriptional regulation of *ATGs* (4). However, the transcriptional regulators and precise mechanisms of autophagy regulation, as well as whether the induction or repression of *ATGs* is associated with autophagy level, are still poorly understood in M. oryzae.

The Sin3 histone deacetylase complex (Sin3-HDAC) has been implicated in the regulation of numerous cellular processes, including development, maintenance of pluripotency, and cell cycle regulation (30–32). The central platform of the complex is the Sin3 protein, which acts as a scaffold protein and master regulator by interacting with DNA-binding factors and recruiting histone deacetylase, such as HDAC1 or HDAC2, to assemble a functional complex (33). Sin3 is termed a coregulator complex that modifies chromatin at both gene-specific and global levels, coordinating with a series of Sin3-associated proteins (31). In yeast, the Sin3-HDAC complex is composed of Rpd3L and Rpd3S, which share common subunits of Sin3, Rpd3, and Ume (32–34). Moreover, the Ume6-Sin3-Rpd3 complex has been reported to regulate the transcription of *ATG8* through direct occupancy by Ume6 (35, 36). In Pichia pastoris, Sin3 is a negative regulator in mitophagy through directly binding to *ATG32* (37). In Caenorhabditis elegans, enhanced autophagy in *sin3* deletion mutants is dependent on reactive oxygen species (ROS) and intracellular oxidative stress (38, 39). However, whether and how Sin3 regulates autophagy of M. oryzae remain unclear.

In our previous study, we found that Sin3-HDAC is required for the normal distribution of trimethylation of lysine 27 on histone H3 (H3K27me3) occupancy and transcriptional silencing in M. oryzae (40). In this study, we revealed that Sin3 acts as a transcriptional repressor of *ATGs* to negatively regulate autophagy formation in M. oryzae. Furthermore, it was confirmed that Sin3 could directly bind to the chromatin of *ATGs* and regulate their levels of histone acetylation. Our data suggest a novel mechanism of Sin3 in the transcriptional regulation of *ATGs* and modulation of autophagy in M. oryzae.

## RESULTS

### Deletion of *SIN3* leads to an increased expression of autophagy-related genes in Magnaporthe oryzae.

Autophagy plays an important role in fungal development and pathogenicity (5). In M. oryzae, autophagy is required for the degradation of the conidial components during appressorium formation (3, 29). In our previous study, Sin3 was found to function primarily as a globally transcriptional repressor in M. oryzae (40). By analyzing the transcriptome sequencing (RNA-seq) data, we noted that expression levels of extensive *autophagy-related genes* (*ATGs*) were significantly upregulated in the Δ*sin3* strain compared with those in the wild-type (WT) strain, which included *ATG1*, *ATG3*, *ATG4*, *ATG8*, *ATG9*, *ATG11*, *ATG12*, *ATG13*, *ATG14*, *ATG15*, *ATG16*, *ATG17*, *ATG23*, *ATG24*, *ATG27*, *ATG26*, and *ATG29* (see Fig. S1 in the supplemental material).

**FIG 1:**
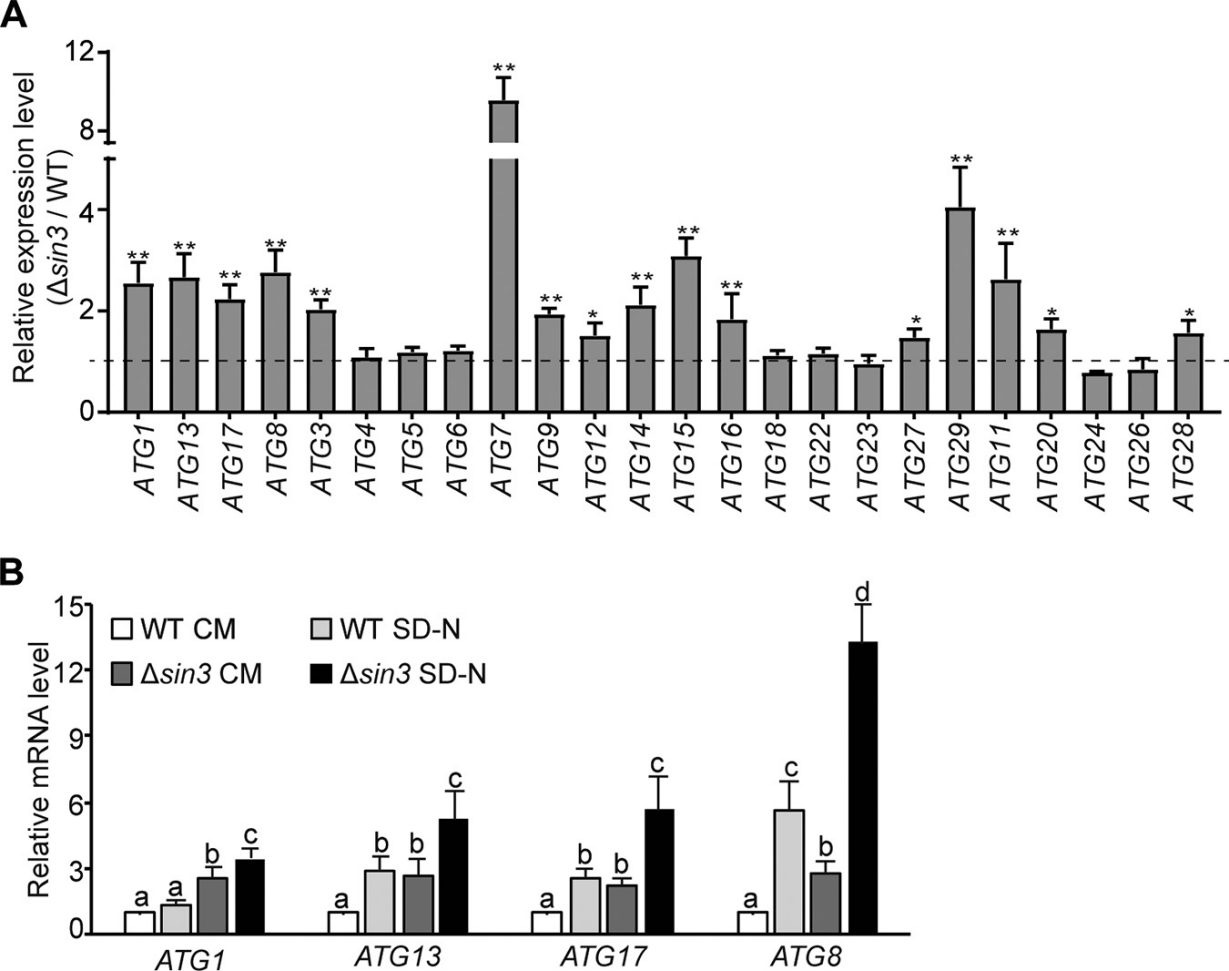
Deletion of *SIN3* increases the transcriptional expression of *autophagy-related* genes (*ATGs*) in Magnaporthe oryzae. (A) Relative expression of *ATGs* in the Δ*sin3* strain compared with those of the WT strain when cultured in the liquid nutrient-rich medium (CM). Values are the average from three biological replicates, and the asterisks indicate the significant difference between the WT and Δ*sin3* strains (*, *P < *0.05; **, *P < *0.01). (B) Relative expression of *ATG1*, *ATG13*, *ATG17*, and *ATG8* in the WT and Δ*sin3* strains cultured under the CM and nutrient-deficient medium (SD-N) conditions. Strains were cultured in the liquid CM for 2 days and then transferred to SD-N for 4 h. Values are the means ± SD from three biological replicates. Different letters (a, b, and c) indicate the significant differences as determined by one-way ANOVA (*P < *0.05).

To validate the RNA-seq data, transcriptional levels of these *ATG*s in the WT and Δ*sin3* strains grown in the liquid nutrient-rich complete medium (CM) were determined by reverse transcriptase quantitative PCR (RT-qPCR). Consistent with results of RNA-seq, expression levels of extensive *ATGs* were significantly upregulated in the Δ*sin3* strain compared with those of the WT strain under the CM conditions. In M. oryzae, Atg1, Atg13, and Atg17 form an initiation complex during autophagy induction (29). It was noteworthy that the stable, elevated expression of *ATG1*, *ATG13*, and *ATG17* was observed in the Δ*sin3* strain (Fig. 1A). To further confirm these results, the expression levels of *ATG1*, *ATG13*, and *ATG17* in the autophagy-inductive nutrient-deficient medium without amino acids and ammonium sulfate (SD-N) were detected. The results showed that the upregulated pattern of *ATG1*, *ATG13*, and *ATG17* was extended in the Δ*sin3* strain after a 4-h exposure in SD-N medium (Fig. 1B). Taken together, these results indicated that Sin3 functions as a transcriptional repressor of *ATGs* in M. oryzae.

### Loss of *SIN3* promotes autophagy in M. oryzae.

**FIG 2:**
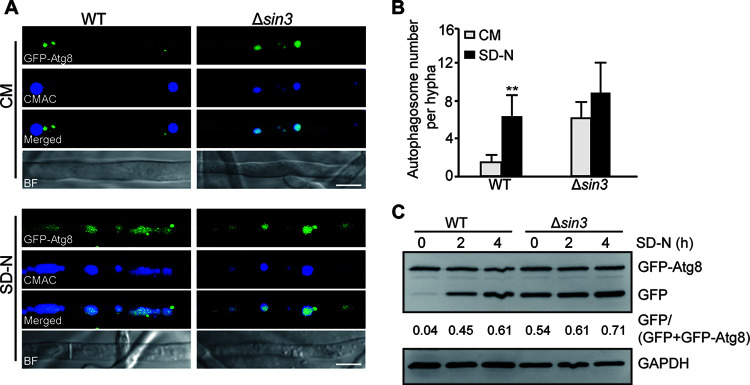
Loss of *SIN3* promotes autophagy in Magnaporthe oryzae. (A) The GFP-Atg8 localization in the *GFP-ATG8* and *Δsin3/GFP-ATG8* strains under liquid nutrient-rich medium (CM) and nutrient-deficient medium (SD-N) conditions. Strains were cultured in the CM for 2 days and then transferred to SD-N for 4 h. Mycelia were stained with CMAC, and images were captured by fluorescence microscopy. Bars, 20 μm. (B) The autophagosome numbers of the *GFP-ATG8* and *Δsin3/GFP-ATG8* strains in the CM and SD-N medium. At least 25 hyphal segments were used to calculate the autophagosomes. Values are means ± SD from three replicates. The asterisks indicate the significant difference between the WT and Δ*sin3* strains (**, *P < *0.01). (C) Immunoblot analysis of the *GFP-ATG8* and *Δsin3/GFP-ATG8* strains under CM and SD-N conditions. The immunoblot of GAPDH was presented as the internal loading control. The degradation rates were calculated with the following formula: GFP/(GFP + GFP-Atg8). Two biological replicates were performed with similar results.

The increased expression of *ATGs* in the Δ*sin3* strain prompted us to speculate that Sin3 might regulate autophagy in M. oryzae. To test this hypothesis, the fusion construct of green fluorescent protein (*GFP*)-*Atg8*, which is a widely used marker of autophagy activity, was introduced into the WT and Δ*sin3* strains (3). The resulting *GFP-ATG8* and Δ*sin3*/*GFP-ATG8* strains were grown in CM for 2 days and then transferred to the SD-N medium for additional 4 h to induce autophagy. To visualize the vacuoles, 7-amino-4-chloromethylcoumarin (CMAC) staining was performed before microscopic observation with a confocal microscope. In the CM, the fluorescence signal in the *GFP-ATG8* strain was located mainly in the punctate structures in cytosol and rarely in the vacuoles, while the fluorescence signal in the Δ*sin3*/*GFP-ATG8* strain was located predominantly in vacuoles (Fig. 2A). Under SD-N conditions, the fluorescence signal of GFP-Atg8 in the *GFP-ATG8* strain was presented not only in the cytosol but also in the vacuoles which were accompanied with induced autophagy. However, the fluorescence of GFP-Atg8 in the Δ*sin3*/*GFP-ATG8* strain was almost shown in the vacuoles under autophagy-inductive conditions (Fig. 2A), which is obviously different with that in the *GFP-ATG8* strain. An monodansylcadaverine (MDC) staining assay further showed that more autophagosomes were observed in the Δ*sin3* strains than that in the WT on CM (see Fig. S2 in the supplemental material). Furthermore, the number of autophagosomes in the Δ*sin3*/*GFP-ATG8* and *GFP-ATG8* strains were counted by fluorescence microscopy under normal and autophagy-inductive conditions. Compared with the *GFP-ATG8* strain, the number of autophagosomes increased significantly in the Δ*sin3*/*GFP-ATG8* strain (Fig. 2B).

Subsequently, we determined the level of autophagy by detecting GFP signal with Western blotting. Under the CM conditions, a significantly stronger band of free GFP was detected in the Δ*sin3*/*GFP-ATG8* strain compared with that in the *GFP-ATG8* strain (Fig. 2C), which indicated a higher level of autophagy in the Δ*sin3*/*GFP-ATG8* strain. After 2 days growth in the CM conditions, the detected strains were transferred to the SD-N medium for further autophagy assays. The signal of free GFP was still stronger than that in the CM medium, as more fusion protein of GFP-Atg8 was degraded into free GFP (Fig. 2C). When calculated with ratio of free GFP to the sum of GFP-Atg8 and free GFP, the Δ*sin3*/*GFP-ATG8* strain had an extremely higher ratio of free GFP to total GFP fusion proteins under the CM conditions, further implying the higher level of autophagy in the Δ*sin3*/*GFP-ATG8* strain than that in the *GFP-ATG8* strain (Fig. 2C). Taken together, these results indicated that a loss of *SIN3* promotes autophagy in M. oryzae.

### Sin3 negatively regulates transcriptional expression of *ATGs* through direct occupancy and histone deacetylation in M. oryzae.

The molecular mechanism mediated by the histone deacetylation complex (HDAC) has two layers of regulation (10). One layer is regulating the transcriptional expression of target genes at the transcriptional level and the other is regulating the acetylation of the target protein at the posttranslational level (41). In our recent study, the global level of acetylation of histone H4 lysine 16 (H4K16ac), which has been reported to be involved in the regulation of autophagy (6), was increased in the Δ*sin3* strain (40).

**FIG 3:**
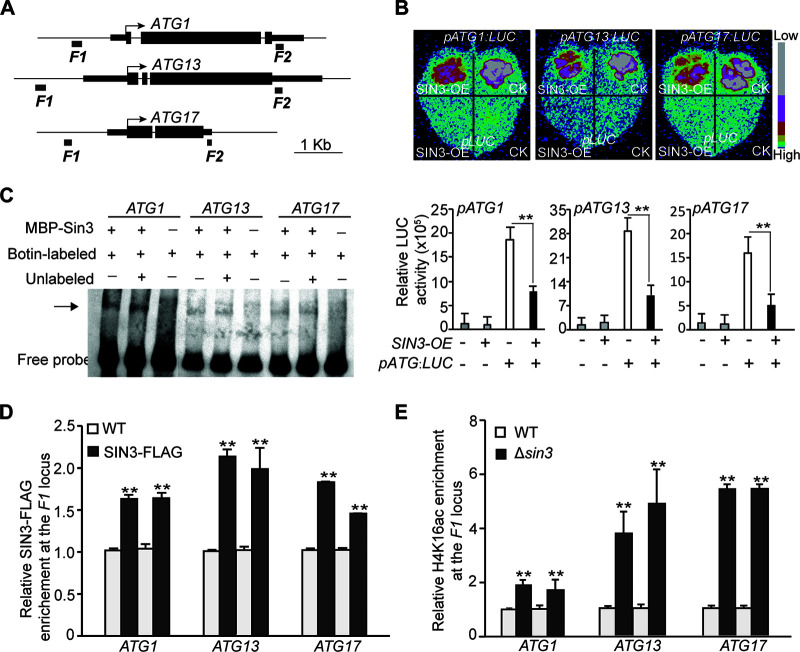
Sin3 negatively regulates the transcription of *autophagy-related* genes in Magnaporthe oryzae. (A) Schematic representation of genomic regions of *ATG1*, *ATG13*, and *ATG17* genes that were examined. (B) Transient luciferase (LUC) assays showing that the overexpression of *SIN3* negatively regulates transcription of *ATG1*, *ATG13*, and *ATG17*. Representative images of N. benthamiana leaves are shown to indicate the relative LUC activity between different combinations at the top. Values are means ± SD from three replicates, and the asterisks indicate the significant difference (**, *P < *0.01). (C) EMSA indicated the binding of Sin3 to the loci of *ATG1*, *ATG13*, and *ATG17*. The unlabeled probes for the competition assay were added as 20-fold excess. (D) ChIP-qPCR analysis showing the relative enrichment of Sin3-FLAG in the *F1* locus of *ATG1*, *ATG13*, and *ATG17* in the WT and *SIN3-FLAG* strains. Mycelia cultured in the liquid CM were collected for ChIP experiments with the FLAG antibody. The relative fold of enrichment in the *SIN3-FLAG* strain over that of the WT strain with two independent replicates is shown. Values are means ± SD from three technical replicates. The asterisks indicate the significant difference between the WT and *SIN3-FLAG* strains (**, *P < *0.01). (E) ChIP-qPCR analysis showing the relative enrichment of H4K16ac in the *F1* locus of *ATG1*, *ATG13*, and *ATG17* in the WT and Δ*sin3* strains. Mycelia cultured in the liquid CM were collected for ChIP experiments with the H4K16ac antibody. The relative enrichment in the Δ*sin3* strain over that in the WT strain with two independent replicates is shown. Values are means ± SD from three technical replicates.

To investigate whether Sin3 regulates the transcription of *ATGs*, transient luciferase assays were conducted. The expression of luciferase driven by the promoter of *ATG1*, *ATG13*, and *ATG17* individually was transiently repressed by the overexpression of *SIN3* (Fig. 3A and B), suggesting that Sin3 might directly regulate the transcription of *ATGs*. Moreover, an electrophoretic mobility shift assay (EMSA) showed that the recombinant Sin3 protein can directly bind to these loci (Fig. 3C; see Fig. S3 in the supplemental material). To examine whether Sin3 could directly bind to the chromatin regions of these genes *in vivo*, the *SIN3-FLAG* strain was created in the Δ*sin3* background. The resultant *SIN3-FLAG/*Δ*sin3* strain could fully complement the phenotypic defects of the Δ*sin3* strain, indicating that the FLAG-tagged Sin3 protein is functional (see Fig. S4A and D in the supplemental material). Furthermore, a chromatin immunoprecipitation followed by quantitative PCR (ChIP-qPCR) assay revealed that compared with the WT strain, the examined regions at the promoters of *ATG1*, *ATG13*, and *ATG17* had 1.5- to 2-fold enrichment in the *SIN3-FLAG/*Δ*sin3* strain (Fig. 3C). In contrast, no obvious enrichment of Sin3 in the 3′ untranslated regions of these genes was detected in the *SIN3-FLAG/*Δ*sin3* strain compared with those in the WT strain (Fig. S4E). Next, we asked whether direct occupancy on the *ATGs* by Sin3 would associate with the changed levels of histone acetylation. To answer this question, a ChIP-qPCR assay with an anti-H4K16ac antibody was performed with the Δ*sin3* and WT strains. Compared with the WT strain, the enrichments of H4K16ac at the promoter regions of *ATG1*, *ATG13*, and *ATG17* in the Δ*sin3* strain were 2- to 6-fold higher than that in the WT strain (Fig. 3D). Together, we concluded that the loss of *SIN3* directly dissociates the occupancy from those *ATGs* and results in histone hyperacetylation at the chromatin of *ATGs*.

### Autophagy induction resulted in downregulated expression of *SIN3*.

**FIG 4:**
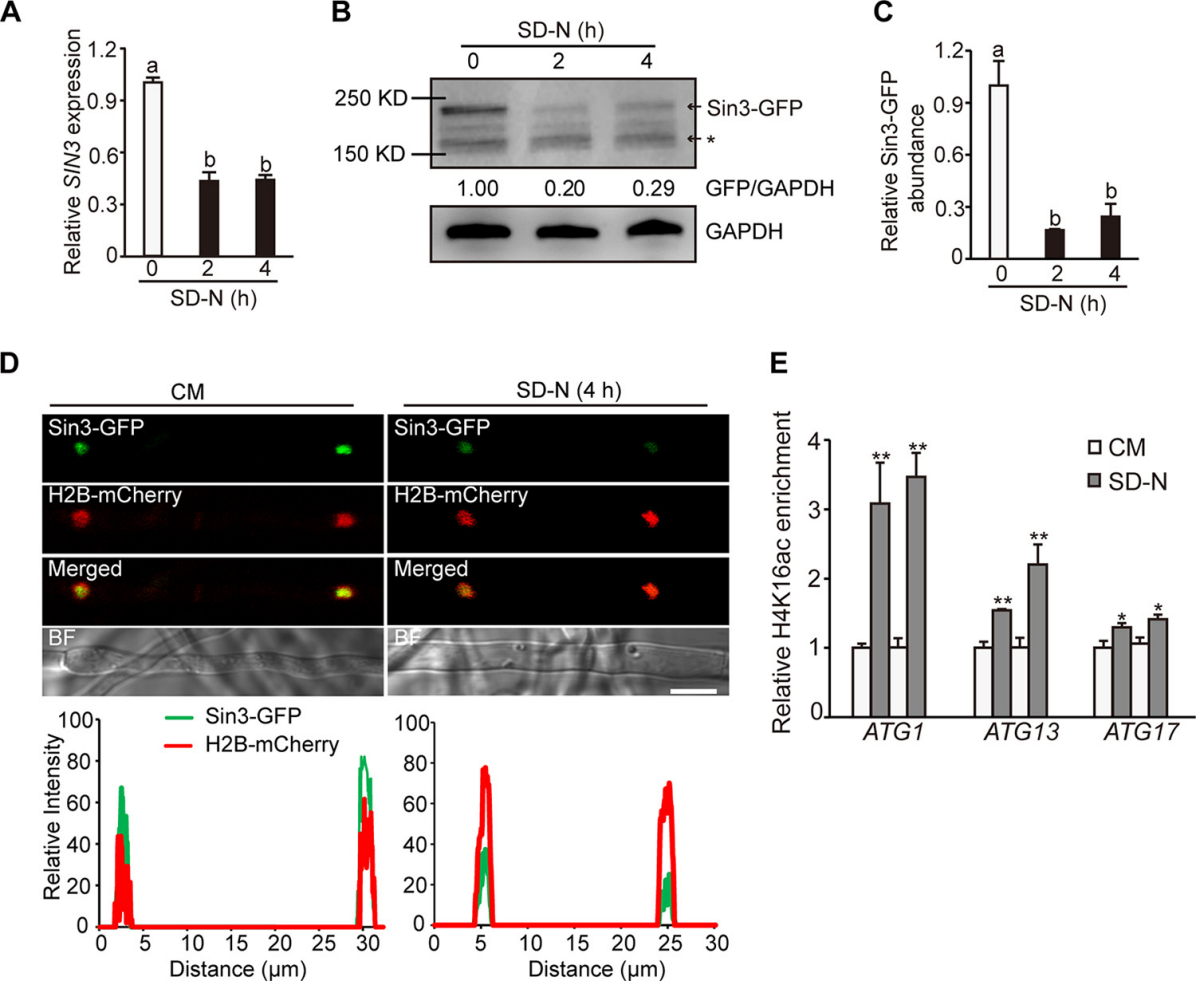
Transcriptional expression and protein accumulation of Sin3 were decreased during autophagy induction. (A) The expression level of *SIN3* in the indicated treatment of the WT strain. Strains were cultured in the CM for 2 days and transferred to the SD-N medium for an additional 2 and 4 h. Values are means ± SD from three biological replicates. Different letters (a or b) indicate significant differences tested by one-way ANOVA (*P < *0.01). (B and C) Relative abundance of Sin3-GFP detected by immunoblot analysis in the indicated treatment of the Δ*sin3*-C strain. The main band (top) of Sin3-GFP is estimated to be 190 kD (molecular weight for protein). The asterisk indicates the unspecific band. Values are means ± SD from three biological replicates. Different letters (a or b) indicate significant differences tested by one-way ANOVA (*P < *0.01). (D) The localization and relative abundance of Sin3-GFP in mycelia with indicated culture conditions. Images were photographed by a confocal microscope. The relative intensities of Sin3-GFP and H2B-mCherry were analyzed by ImageJ software. Bar, 5 μm. (E) ChIP-qPCR analysis showing H4K16ac enrichment on the *F1* region of *ATG1*, *ATG13*, and *ATG17*. Mycelia cultured in the liquid CM and SD-N medium were collected for ChIP experiments with the anti-H4K16ac antibody. The relative enrichment of H4K16ac in the WT strain from the SD-N medium over that from the CM medium with two independent replicates is shown. Values are means ± SD from three biological replicates. The asterisks indicate the significant difference of the enrichment of H4K16ac between the CM and SD-N growth conditions (*, *P < *0.05; **, *P < *0.01).

Transcriptional expression of *SIN3* was further examined under the autophagy-induced conditions. As shown in Fig. 4A, the relative expression of *SIN3* was significantly decreased compared with that under the CM condition. To determine whether the decreased transcription would result in the reduced abundance of the Sin3 protein, the complementary strain (Δ*sin3*-C) was created, in which *SIN3* was driven by its native promoter and fused with GFP. Immunoblot analysis showed that the relative intensity of Sin3-GFP was also decreased under SD-N conditions compared with that grown in the CM (Fig. 4B and C; see Fig. S5 in the supplemental material). Next, the protein abundance and the cellular localization of Sin3 were detected by analyzing the fluorescent intensity of the GFP fusion signal in the Sin3-GFP strain. A live cell epifluorescence observation showed that Sin3-GFP colocalized with H2B-mCherry in the nucleus when growing in both CM and SD-N medium (Fig. 4D). However, the relative fluorescent intensity of Sin3-GFP was obviously attenuated under SD-N conditions compared with that in the CM. Consistent with epifluorescence observation, hyperacetylation of H4K16 was detected at the promoter of *ATGs* when the strains were transferred to the SD-N medium compared with that in the CM (Fig. 4E). Together, these results suggested that both transcriptional expression and protein accumulation of Sin3 are significantly decreased during autophagy induction and that the decreased occupancy of Sin3 from *ATGs* would result in histone hyperacetylation and increased expression of *ATGs* and in turn promote autophagy under nutrient-deficient conditions.

### Constitutive expression of *ATG1*, *ATG13*, and *ATG17* promotes autophagy in M. oryzae.

**FIG 5:**
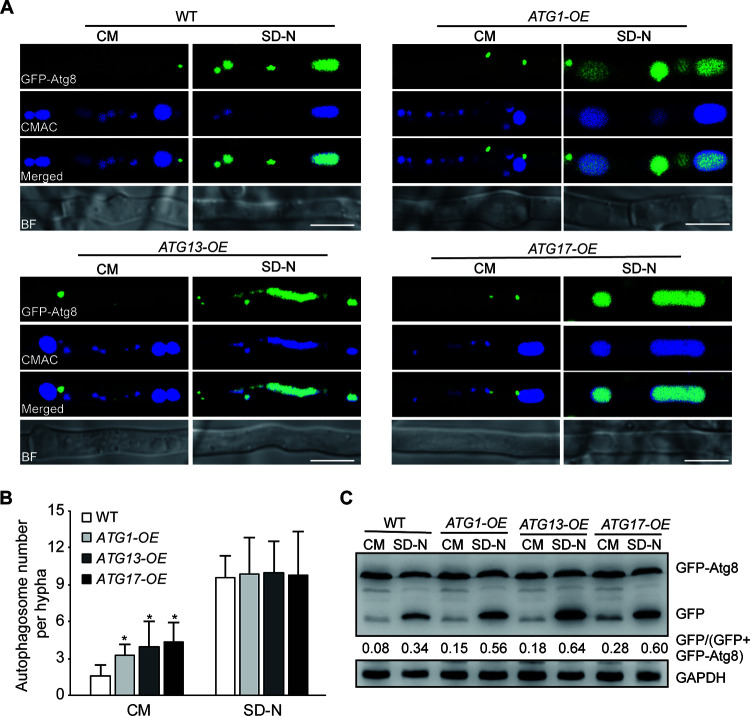
Constitutive expression of *ATG1*, *ATG13*, and *ATG17* promotes autophagy in Magnaporthe oryzae. (A) The GFP-Atg8 localization in the *GFP-ATG8*, *ATG1-OE/GFP-ATG8*, *ATG13-OE/GFP-ATG8*, and *ATG17-OE/GFP-ATG8* strains in CM and SD-N medium. Strains were cultured in CM for 2 days and then transferred to SD-N medium for 4 h. Mycelia were stained with CMAC and then photographed with a confocal microscope. BF, bright field. Bars, 5 μm. (B) The autophagosome numbers in the *GFP-ATG8*, *ATG1-OE/GFP-ATG8*, *ATG13-OE/GFP-ATG8*, and *ATG17-OE/GFP-ATG8* strains under CM and SD-N conditions. At least 25 hyphal segments were used to calculate the number of autophagosomes. Values are means ± SD from three technical replicates. The asterisks indicate the significant difference between the WT and *ATG-OE* strains (*, *P < *0.05). (C) Immunoblot analysis of GFP in the *GFP-ATG8*, *ATG1-OE/GFP-ATG8*, *ATG13-OE/GFP-ATG8*, and *ATG17-OE/GFP-ATG8* strains under the CM and SD-N conditions. The immunoblot of GAPDH was presented as the internal loading control. The degradation rates were calculated with the following formula: GFP/(GFP + GFP-Atg8). Three biological replicates were performed with similar results.

In the process of autophagy, Atg17-Atg13 complex formation plays an important role in autophagosome formation via activating the Atg1 kinase (29). To determine whether the increased expression of *ATG1*, *ATG13*, and *ATG17* could promote autophagy in M. oryzae, strains with constitutive expression of *ATG1*, *ATG13*, and *ATG17* (*ATG1-OE*, *ATG13-OE*, and *ATG17-OE*), respectively, were created in the *GFP-ATG8* background (see Fig. S6A in the supplemental material). In the CM, the fluorescence signal of GFP-Atg8 was located mostly in the punctate structures in the cytosol and rarely in the vacuoles in the *GFP-ATG8* strain. In contrast to the distribution of GFP in the WT strain, the GFP-Atg8 was localized partly in the vacuole in the *ATG1-OE*, *ATG13-OE*, and *ATG17-OE* strains (Fig. 5A). Furthermore, the numbers of autophagosomes were counted under autophagy-inductive condition. Compared with the WT strain, the number of autophagosomes was increased in the *ATG1*-OE, *ATG13*-OE, and *ATG17*-OE strains (Fig. 5B). Meanwhile, a greater abundance of free GFP was detected clearly in the *ATG1*-OE, *ATG13*-OE, and *ATG17*-OE strains than that in the WT strain (Fig. 5C; Fig. S6B and C), indicating that the constitutive expression of *ATG1*, *ATG13*, and *ATG17* leads to a higher level of autophagy under normal and autophagy-inductive conditions. Taken together, these results further suggested that the increased expression of *ATG1*, *ATG13*, and *ATG17* in the Δ*sin3* strain associates with a higher level of autophagy in M. oryzae.

### Sin3 is necessary for mycelial growth, conidiation, and appressorium formation in M. oryzae.

**FIG 6:**
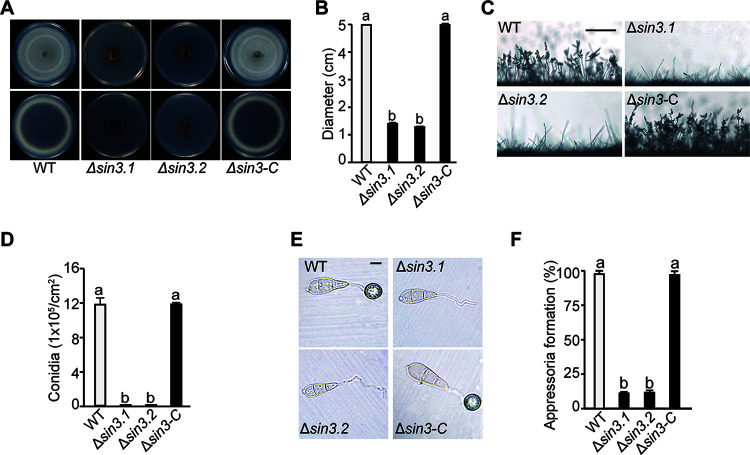
Sin3 is required for the fungal growth, conidiation, and appressoria formation in Magnaporthe oryzae. (A and B) Radical growth and statistical analysis of the indicated strains grown in the CM for 7 days. Both the top and bottom of colonies were imaged. Values are the means ± SD from three biological replicates. Different letters (a or b) indicate significant differences tested by one-way ANOVA (*P < *0.05). (C) The conidiophore morphology of the indicated strains. Strains were cultured in CM for 7 days to observe the conidiophores. Bar, 200 μm. (D) Statistical analysis of conidiation of the indicated strains in the CM. Values are means ± SD from three biological replicates. Different letters (a or b) indicate the significant differences as determined by one-way ANOVA (*P < *0.05). (E) The appressoria formation of the indicated strains. Strains were cultured in CM for 7 days, and then the conidia were collected to induce appressoria on the hydrophobic surface for 16 h. Bar, 20 μm. (F) The appressoria formation rates of the indicated strains. Values are means ± SD from three biological replicates. Different letters (a or b) indicate the significant differences determined by one-way ANOVA (*P < *0.05).

**FIG 7:**
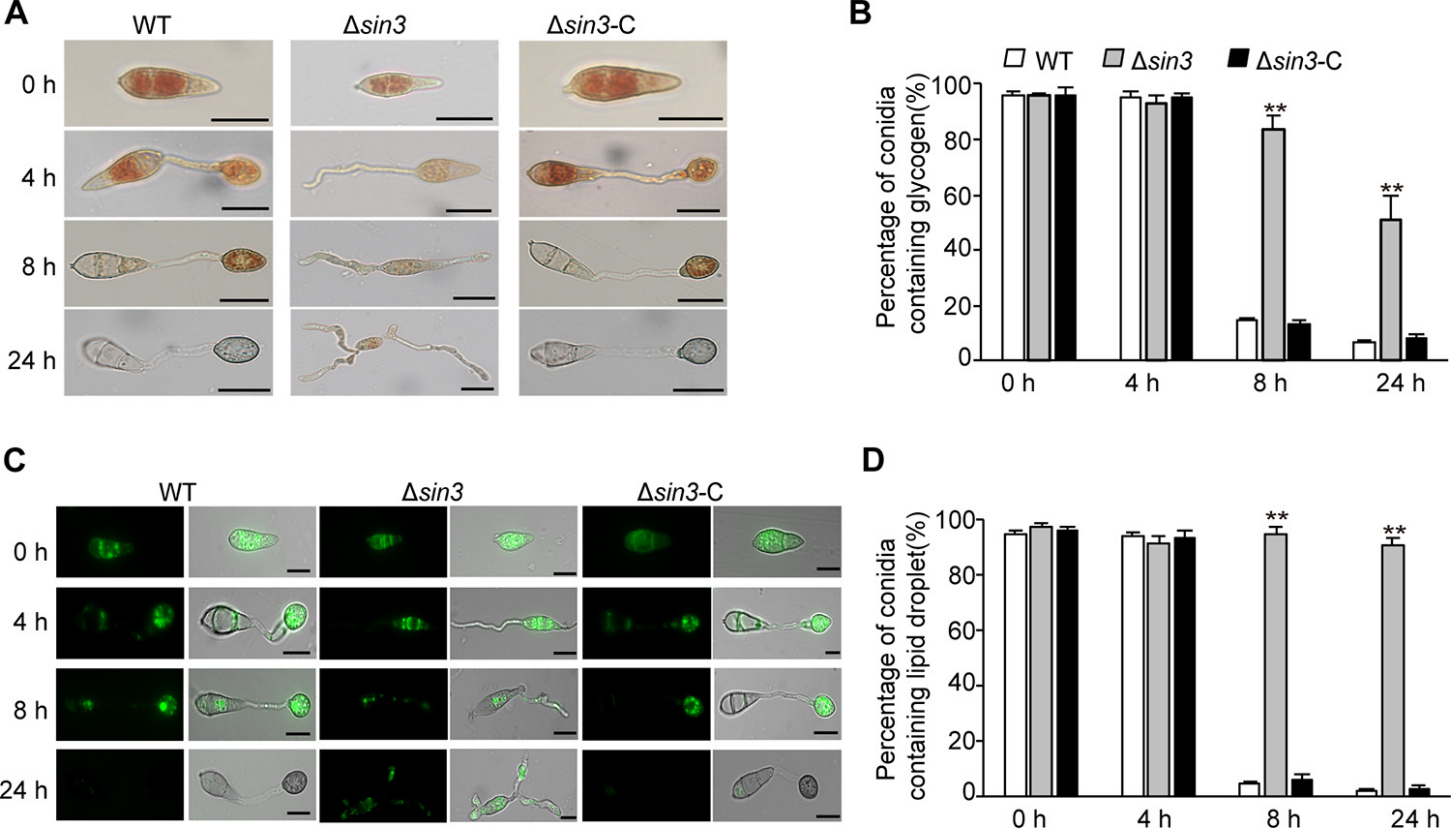
Glycogen degradation and lipid droplet translocation were delayed in the Δ*sin3* strain during appressoria formation. (A and B) Observation and quantification of conidia containing glycogen at 0, 4, 8, and 24 hpi. Conidial suspensions were inoculated on the hydrophobic surfaces and stained with I_2_/KI solution. Bars, 10 μm. (C and D) Observation and quantification of conidia containing lipid droplets at 0, 4, 8, and 24 hpi. Conidial suspensions were inoculated on the hydrophobic surfaces and stained with boron-dipyrromethene (BODIPY) solution. Bars, 10 μm.

To determine whether Sin3 is required for fungal growth and development, the WT, Δ*sin3*, and complementary strains were cultured on the CM or 7 days. The results showed that the Δ*sin3* strain formed smaller colonies that were around 14 mm in diameter, while the WT and complementary strain showed normal colonies that were nearly 50 mm in diameter (Fig. 6A and B). Moreover, the statistics of spores revealed that the number of conidia in the Δ*sin3* strain was largely decreased, compared with that in the WT and complementary strains (Fig. 6C and D). In addition, the ratio of appressoria formation in the Δ*sin3* strain was greatly reduced to only 10% under the inductive conditions, while the ratio in the WT strain was nearly 100% (Fig. 6E and F). Furthermore, glycogen degradation and lipid droplet translocation, which are crucial for appressorium formation, were observed. In contrast to complete glycogen degradation and lipid translocation in the WT strain, glycogen and lipid droplets in the conidia of Δ*sin3* were still retained even at 24 hours postinoculation (hpi) (Fig. 7A to D). These results showed that both glycogen degradation and lipid droplet translocation were delayed severely in the Δ*sin3* strains compared with those of the WT. These results implied that Sin3 is necessary for mycelial growth, conidiation, and appressorium formation in M. oryzae.

### Sin3 is required for virulence in M. oryzae.

**FIG 8:**
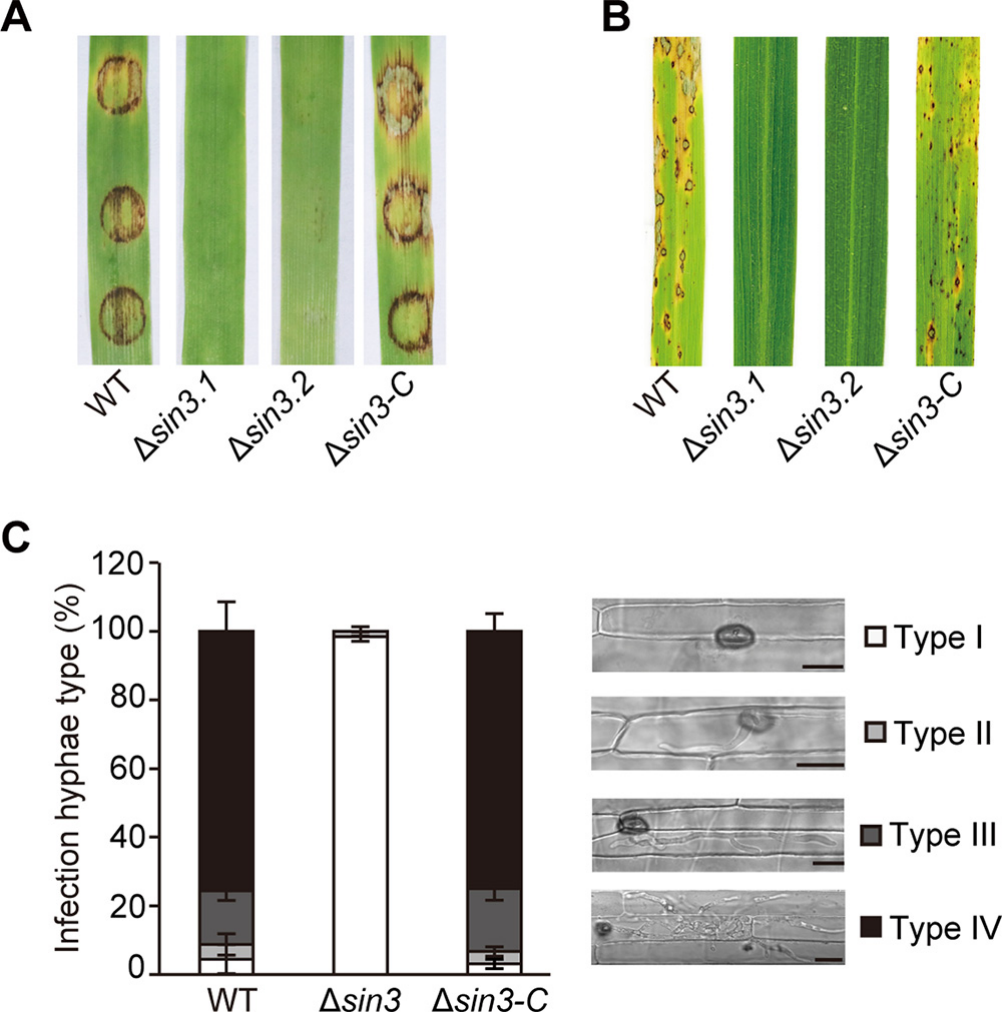
Sin3 is required for the virulence of Magnaporthe oryzae. (A) BLAST infection assays on the barley leaves inoculated with mycelial plugs from the indicated strains for 4 days. Representative inoculated leaves were shown. (B) Rice seedling assays were used to evaluate pathogenicity. The conidial suspensions (5 × 10^4^ conidia/mL) from the indicated strains and 21-day-old rice seedlings were used for spraying inoculation. Representative inoculated leaves were shown. (C) Observation and statistical analysis of invasive hyphae growth in rice sheath cells at 40 hours postinoculation (hpi). Four types of invasive hyphae (illustrated at the right with the corresponding column) were quantified as no penetration, penetration with primary hyphae, penetration with secondary invasive hyphae in the first invaded cell, and invasive hyphae spreading into neighboring cells. Data represent the means ± SD from three independent repeats, with over 100 appressoria per analysis. Bars, 10 μm.

To investigate whether the disruption of *SIN3* affects the pathogenicity of M. oryzae, infection assays were performed with susceptible barley and rice seedlings. For the barley leaf infection assay, mycelial plugs from the WT, Δ*sin3*, and complementary strains were inoculated on the detached barley leaves. Four days after inoculation, no visible lesion was formed on the Δ*sin3*-inoculated leaves, while large lesions were formed on the leaves inoculated with the WT and complementary strains (Fig. 8A). Furthermore, the 5 × 10^4^/mL conidial suspensions were used for spray inoculation on 3-week-old susceptible rice seedlings. Consistent with the results of the barley leaf infection assay, few lesions developed on the Δ*sin3*-inoculated leaves at 7 days postinoculation (dpi), while the typical spindle-shaped blast lesions were formed on the leaves inoculated with the WT and complementary strains (Fig. 8B). Taken together, we concluded that Sin3 is required for the virulence of M. oryzae.

To further explore how Sin3 affects virulence, a rice sheath infection assay was performed with inoculation of the WT, Δ*sin3*, and complementary strains. Consistent with the results on the hydrophobic surfaces, the absolute majority of conidia could not form appressoria of the Δ*sin3* strain. Furthermore, four types of invasive hyphae formed by appressoria were quantified at 40 hpi. Nearly 95% of appressoria of the WT and complementary strains successfully infected leaf sheath cells, while almost all appressoria of the Δ*sin3* strain failed to infect leaf sheath cells (Fig. 8C). These results suggested that the loss of pathogenicity in the Δ*sin3* strain is due to the crucial role of Sin3 in the appressoria formation and penetration.

## DISCUSSION

**FIG 9:**
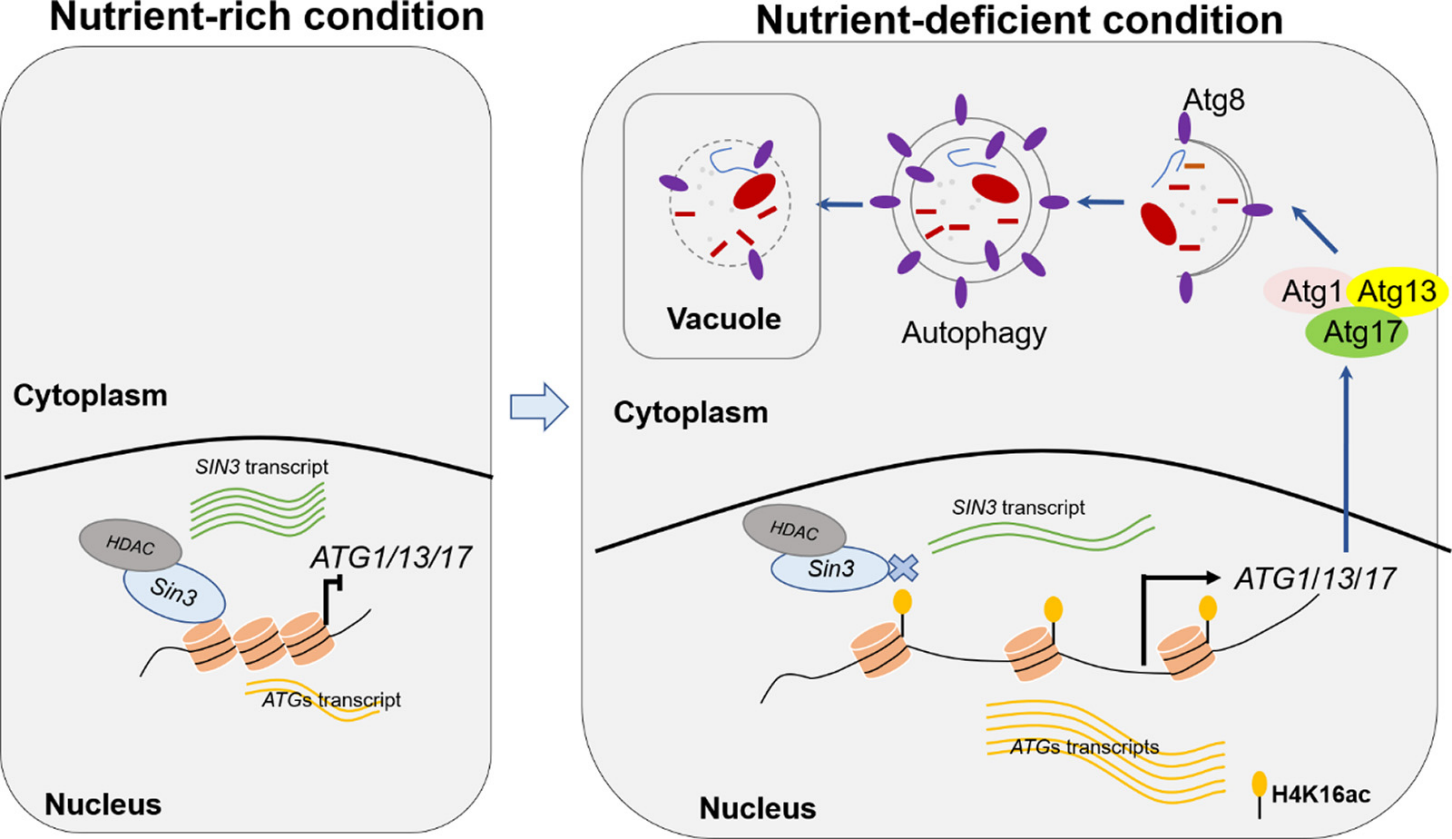
A model of Sin3 in the transcriptional regulation of *ATG* genes under the nutrient-rich and -deficient conditions. Under the nutrient-rich conditions, Sin3 inhibits autophagy with a basal level through directly repressing the transcription of *ATG1-ATG13-ATG17* genes. Upon nutrient-deficient treatment, the transcriptional level of *SIN3* decreases and the dissociation of Sin3 from those *ATG* genes associates with histone hyperacetylation and activates their transcriptional expression and in turn contributes to autophagy induction.

An appropriate status of autophagy is crucial to organisms and is tightly regulated both temporally and continuously. Transcriptional regulation of *autophagy-related genes* is an important layer in the autophagy regulation, but the transcriptional regulators are poorly identified, especially in plant fungal pathogens. In this study, we identified a novel regulator, of which Sin3, a component of histone deacetylation complex, epigenetically regulates transcriptional expression of *ATGs* to repress autophagy induction in M. oryzae (Fig. 9).

The expression of many *ATGs* as well as the level of their encoding proteins are substantially increased in response to autophagy induction, while the lower level of ATG proteins is adequate for the cytoplasm-to-vacuole targeting pathway and the basal level of autophagy (2). Prompt upregulation of *ATG* expression upon autophagy induction is critical for optimal autophagic efficiency. In response to inducing autophagy, the short-term transcriptional response helps cells to survive and adapt to adverse environmental stresses properly, while the long-term transcriptional responses to autophagy, such as constitutive expression of *ATGs*, would be a challenge for energy homeostasis and development (42). However, the mechanism of induction or activation of *ATGs* is largely unknown and very few transcriptional regulators were identified, especially in plant fungal pathogens. In our study, an increased expression of *ATG1*, *ATG13*, and *ATG17* in the Δ*sin3* strain was accompanied with the increased enrichment of H4K16ac on their promoters. During autophagy induction, the decreased level of Sin3 releases the occupancy from *ATGs* and leads to their increased expression and further contributes to autophagy induction under nutrient-deficient conditions. However, whether the deletion of those *ATGs* in the Δ*sin3* strain could restore the prematured autophagy is worthy for further exploration. Thus, the finding of histone modifications and epigenetic regulators, which are tightly associated with the expression of *ATGs* and thereafter autophagic process, may offer a framework to understand the short-term and long-term transcriptional response to stimuli eliciting autophagy.

Sin3 usually acts as scaffold protein to physically associate with DNA-binding factors, a series of Sin3-associated proteins, and histone deacetylase to assemble a functional complex (33). In yeast, Ume6-Sin3-Rpd3 has been reported to work together and Ume6 functions as transcriptional factor. Moreover, Ume6 could directly bind to the chromatin of *ATG8* and *ATG32* and regulate their transcription depending on the presence of Rpd3 and Sin3 (35, 36). However, Ume6 was not conserved among fungal species (36). In M. oryzae, our recent data indicated that Sin3 is physically associated with histone deacetylase Hos2 and Rpd3 (40). Furthermore, we found that Hos2 also negatively regulates autophagy like Sin3 (see Fig. S7 in the supplemental material). These results further implied that histone hyperacetylation in the *ATG* loci of the Δ*sin3* deletion mutant was caused by the dissociation of Sin3-HDAC. In addition, the weakening signal of Sin3-GFP under nitrogen starvation conditions might be due to the sequestration of Sin3-GFP into the nucleolus or degradation. It has been reported previously that Sin3A could be targeted to the nucleolus by interacting with SAP30L in mammals (43). Recently, MoCti6, a potential component of Rpd3-HDAC, was also reported to be involved in the regulation of autophagy (23). So far, no transcriptional factors which have the capability to bind DNA were identified to be involved in the Sin3-Hos2 complex and in the regulation of autophagy. However, genome-wide studies have shown that Sin3 itself has the property as a general transcription factor and predominantly binds within the transcriptional start sites to directly bind to *ATG32* and negatively regulate mitophagy in Pichia pastoris (33, 37). In our study, we also revealed that Sin3 has the capability to directly bind to the chromatin of *ATGs* and negatively regulate the transcriptional expression of extensive *ATGs*, including *ATG1*, *ATG13*, and *ATG17*. Consistent with our results, Sin3 negatively regulates the transcriptional level of *ATG8* and *ATG32* in yeast (37). The repressive role of Sin3 would keep autophagic transcription at a basal level when the process is suppressed, and its negative effect is released on *ATGs* by the starvation-dependent induction of autophagy.

In M. oryzae, several studies have reported that some *ATGs* are required for fungal development and pathogenicity (3, 22, 26, 28). Elimination of autophagy by deleting *ATGs* in plant-pathogenic fungi significantly impairs fungal development and virulence (3). Recent studies found that epigenetic regulators are involved in autophagy in terms of posttranslation modification through direct interaction with ATG proteins, such as the histone acetyltransferase MoGcn5, MoHat1, and FgGcn5 (44–46). Different from those reported regulators, our studies revealed a novel mechanism by which Sin3 functions as a transcriptional repressor of *ATGs* through direct occupancy and histone modification. However, whether defects in the autophagy formation in the Δ*sin3* strain are directly associated with its abnormal fungal growth and reduced pathogenicity is not known. Therefore, whether *SIN3* is involved in other biological process, such as stress response and mitosis, is worthy to further investigate.

In summary, our results revealed that Sin3 maintains autophagy at a low level through binding the promoter of *ATGs* and regulating their levels of histone acetylation to repress their expression under nutrient-rich conditions. While under the nutrient-deficient conditions, the transcription of *SIN3* is decreased and dissociation of Sin3 from those *ATGs* activates the expression of *ATGs* with histone hyperacetylation and in turn promotes autophagy induction.

## MATERIALS AND METHODS

### Fungal strains.

M. oryzae strain B157 was used as the wild-type (WT) strain for obtaining transformants (47). The Δ*sin3*, Δ*sin3*-C, *GFP-ATG8, and H2B-mCherry* strains were described previously (40, 48, 49). To generate the construct of *SIN3-FLAG*, the coding sequence of *SIN3* was cloned to *pFGL822-pRP27-FLAG*. The confirmed plasmids were introduced into the WT strain or the corresponding deletion mutants by Agrobacterium tumefaciens-mediated transformation (ATMT). Strains and primers used in the experiments were listed in Table S1 and S2 in the supplemental material.

### Fungal growth, conidiation, and infection assay.

For measurements of fungal growth and conidiation, strains were grown in the complete medium (CM) at 25°C for 7 days. The appressorium formation and rice seedling infection assay were conducted as described previously (47). For the barley leaf infection, 7-day-old healthy barley (ZJ-8, Hordeum vulgare) was used. For the observation of invasive hypha, 21-day-old healthy rice seedlings (CO39, Oryza sativa) were used for sheath preparation. Conidial suspensions were inoculated into the rice sheath and incubated in the growth chamber with a photoperiod of 16-h light and 8-h dark at 25°C. For the observation of glycogen, conidial suspensions were inoculated on the hydrophobic surface for 0, 4, 8, and 24 h and stained with I_2_/KI solutions before being photographed (50). The inoculated sheath was trimmed manually and observed by using an Olympus BX53 wide-field microscope at 40 hours post inoculation (hpi). All experiments were repeated three times with at least three replicates in each repeat.

### Western blot analysis.

Fresh mycelia (0.1 g) cultured in the liquid CM for 2 days were harvested for detecting FLAG or GFP-tagged proteins. Total proteins were extracted with lysis buffer (50 mM Tris-HCl [pH 7.4], 150 mM NaCl, 1 mM EDTA, 1% Triton X-100, and 1× fresh protein inhibitor cocktail). The resulting proteins were separated by an SDS-PAGE gel, transferred to a polyvinylidene difluoride (PVDF) membrane, and subsequently detected by immunoblotting with an anti-FLAG (Sigma; A8592) or anti-GFP (Abcam; ab290) antibody. The relative intensity of the immunoblots was quantified with Image J software. To detect the autophagy level, the construct of expressing GFP-Atg8 was introduced into the corresponding strains by ATMT (48). The resulting strains were cultured in the liquid CM for 2 days, and then half of the mycelia were transferred to autophagy-inductive medium (SD-N, 1.7 g yeast nitrogen base without amino acids and 20 g glucose per L) for an additional 2 or 4 h for the autophagic assay. Total proteins were extracted, and GFP-Atg8 was detected by immunoblotting with anti-GFP (Abcam; ab290).

### Live cell epifluorescence observation.

To quantify the number of autophagosomes, the WT and Δ*sin3* strains were cultured in the CM and SD-N medium. At least 25 hyphal segments were calculated. Conidial autophagy was monitored by incubating conidia of strains expressing GFP-Atg8 on the hydrophobic surface for 0, 8, and 16 h as described previously (51). In addition, a MDC staining assay was conducted to demonstrate autophagy activities according to previous studies (52). Mycelia of the WT and Δ*sin3* strains cultured in the CM or SD-N medium were stained with the MDC dye (Beyotime; C3018M) for 10 min before being photographed. The fluorescent signals of GFP-Atg8, Sin3-GFP, and H2B-mCherry were captured by a confocal fluorescence microscope (Zeiss LSM700) with 488-nm and 555-nm laser excitation. Vacuoles were observed with 405-nm laser excitation following 7-amino-4-chloromethylcoumarin (CMAC) staining for 30 min. To monitor the translocation of lipid droplets during appressoria formation, conidial suspensions were inoculated on the hydrophobic surfaces and stained with boron-dipyrromethene (BODIPY) solution (Invitrogen; D-3922) for 0, 4, 8, and 24 h before being photographed with a fluorescence microscope (23). Images were subjected to processing with ImageJ software. The intensities of GFP and mCherry fluorescent signals were analyzed by linescan with ImageJ.

### mRNA expression analysis.

Mycelia cultured in the liquid CM for 2 days were collected, and total RNA was extracted with TRIzol reagent (Invitrogen, USA). Total RNAs were reverse transcribed into cDNA with a commercial kit (Toyobo; FSQ-301) according to the manufacturer’s instruction. RT-qPCR was performed with SYBR green qPCR master mix (Toyobo; QST-100) in the LightCycler480 system (Roche). The constitutively expressed *Tubulin* (MGG_00604) was used as an internal control. Primers used in the experiments were listed and described in Table S2.

### Transient expression assays.

For transient expression assays, the 2-Kb promoter regions of *ATG1*, *ATG13*, and *ATG17* were cloned into the pGWB435 vector individually by the Gateway technology (53). The resultant constructs and *SIN3-FLAG* were cotransformed into Agrobacterium tumefaciens GV3101. Transient LUC expression in Nicotiana benthamiana was performed as described previously (54). Agroinfiltrated leaves were brushed with 0.5 mM luciferin, and the subsequent signals were recorded immediately using a HRPCS5 camera (Photek). Data from three independent biological replicates were collected.

### Protein expression and electrophoretic mobility shift assay.

The full-length coding sequence of Sin3 was fused with MBP and cloned into pMAL-c5X. Subsequently, the fused plasmid was introduced into Escherichia coli strain BL21. The expressed MBP-Sin3 was induced with 1 mM isopropyl β-d-1-thiogalactopyranoside (IPTG), incubated at 28°C for 4 h, and then purified with an amylose resin column (New England BioLabs; E8021V). Probes were synthesized and labeled with biotin. EMSA was performed with the LightShift chemiluminescent EMSA kit (Thermo Fisher Scientific; 20148X) according to the manufacturer’s instructions. Binding reactions contained 1 μL 10× binding buffer, 2 μg recombinant MBP-Sin3 or MBP alone (negative control), and 1 μL biotin-labeled probes or unlabeled probes. In the competition assays, unlabeled probes were provided in 25-fold excess. The binding reaction mixtures were incubated at 28°C for 20 min, separated on 6% native PAGE gels, and transferred onto IMMOBILON-NY^+^ transfer membranes (Millipore’ INYC00010). Signals were detected using the chemiluminescent biotin-labeled nucleic acid detection kit (Beyotime Biotech; D3308). Primers for probes were listed and described in Table S3 in the supplemental material.

### Chromatin immunoprecipitation (ChIP) and ChIP-qPCR.

ChIP experiments with mycelia were conducted as described previously (40). Briefly, mycelia (1 g) were cross-linked with 1% formaldehyde for 20 min and stopped with 125 mM glycine for 5 min at room temperature. After grinding, powers were resuspended in the nuclei isolating buffer (10 mM Tris [pH 8.0], 10 mM sodium butyrate, 400 mM sucrose, 0.1 mM phenylmethylsulfonyl fluoride (PMSF), 5 mM β-mercaptoethanol, and 1× fresh protein inhibitor cocktail). The precipitated nuclei were used to extract chromatin with 1 mL lysis buffer (50 mM HEPES [pH 7.5], 150 mM NaCl, 1 mM EDTA, 10 mM sodium butyrate, 0.1% deoxycholate, 0.1% SDS, 1% Triton X-100, 1 mM PMSF, and 1× fresh protein inhibitor cocktail). The obtained chromatin was sonicated with a Diagenode bioruptor (high setting, 16 cycles), and 20 μL chromatin was used as an input DNA extraction. The remainder was precleared with 10 μL protein A Dynbeads (Thermofisher; 10001D) for 1 h and subsequently incubated with anti-FLAG (Sigma, F7425) or anti-H4K16ac (Abcam, ab109463) for 8 h. Another 20 μL protein A Dynbeads was used to capture the protein-DNA mixture. The recovered DNA was used as the templates for the following ChIP-qPCR. ChIP-qPCR assays were performed with two independent repeats. The relative enrichment of the examined fragments was calculated with the reference gene *TUB5* using qPCR. Primers were listed and described in Table S2.

### Statistical analysis.

One-way analysis of variance (ANOVA) was conducted with SigmaStat 3. The two-tailed *t* test was calculated with Excel software.

### Data availability.

All data generated or analyzed during this study are available from the corresponding author on reasonable request.
