# Supplementary material for: Transcriptional Regulation of Autophagy-Related Genes by Sin3 Negatively Modulates Autophagy in Magnaporthe oryzae
Source: Microbiol Spectr. 2023 May 16;11(3):e00171-23. doi: 10.1128/spectrum.00171-23 (PMC10269650; doi:10.1128/spectrum.00171-23)
Supplement: Supplemental file 1 — Fig. S1. Download spectrum.00171-23-s0001.pdf, PDF file, 0.2 MB [file spectrum.00171-23-s0001.pdf]

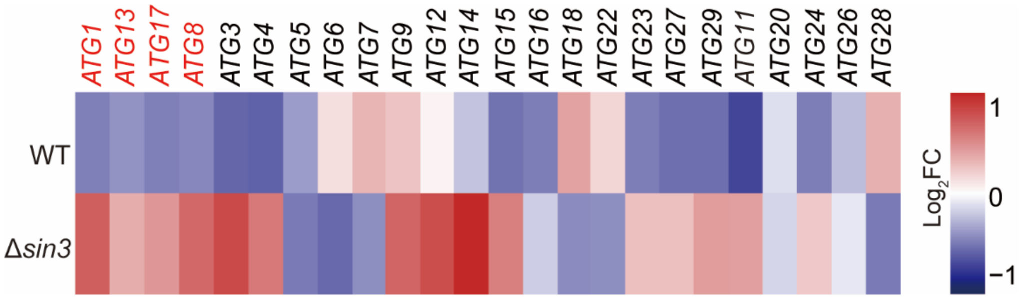

Fig. S1 Heatmap of expressional level of *ATGs* in the WT and Δ*sin3* strains. Relative expression of *ATGs* were extracted from RNA-seq data.
