# Supplementary material for: Transcriptional Regulation of Autophagy-Related Genes by Sin3 Negatively Modulates Autophagy in Magnaporthe oryzae
Source: Microbiol Spectr. 2023 May 16;11(3):e00171-23. doi: 10.1128/spectrum.00171-23 (PMC10269650; doi:10.1128/spectrum.00171-23)
Supplement: Supplemental file 2 — Fig. S2. Download spectrum.00171-23-s0002.pdf, PDF file, 0.1 MB [file spectrum.00171-23-s0002.pdf]

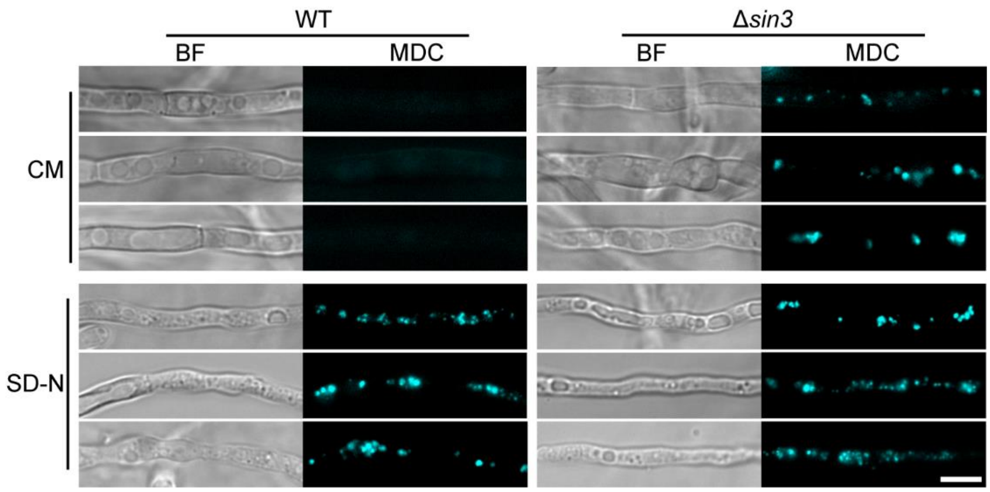

Fig. S2 MDC staining in the indicated strains with or without autophagy induction. Hyphae of the indicated strains were subjected to nitrogen starvation and stained with monodansylcadaverine (MDC) for 10 min before captured. Bar, 5μm.
