# Supplementary material for: Transcriptional Regulation of Autophagy-Related Genes by Sin3 Negatively Modulates Autophagy in Magnaporthe oryzae
Source: Microbiol Spectr. 2023 May 16;11(3):e00171-23. doi: 10.1128/spectrum.00171-23 (PMC10269650; doi:10.1128/spectrum.00171-23)
Supplement: Supplemental file 3 — Fig. S3. Download spectrum.00171-23-s0003.pdf, PDF file, 0.1 MB [file spectrum.00171-23-s0003.pdf]

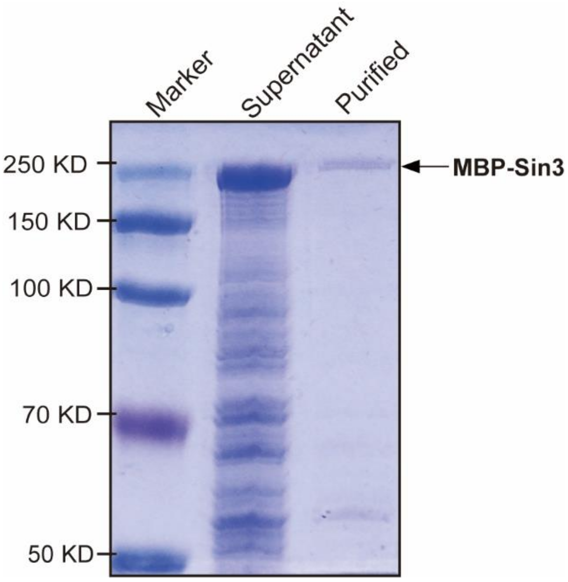

Fig. S3 Protein expression of MBP-Sin3 in *E. coli*. The expressed MBP-Sin3 was induced with 1 mM IPTG and incubated at 28°C for 4 h, and then purified.
