# Supplementary material for: Transcriptional Regulation of Autophagy-Related Genes by Sin3 Negatively Modulates Autophagy in Magnaporthe oryzae
Source: Microbiol Spectr. 2023 May 16;11(3):e00171-23. doi: 10.1128/spectrum.00171-23 (PMC10269650; doi:10.1128/spectrum.00171-23)
Supplement: Supplemental file 4 — Fig. S4. Download spectrum.00171-23-s0004.pdf, PDF file, 0.1 MB [file spectrum.00171-23-s0004.pdf]

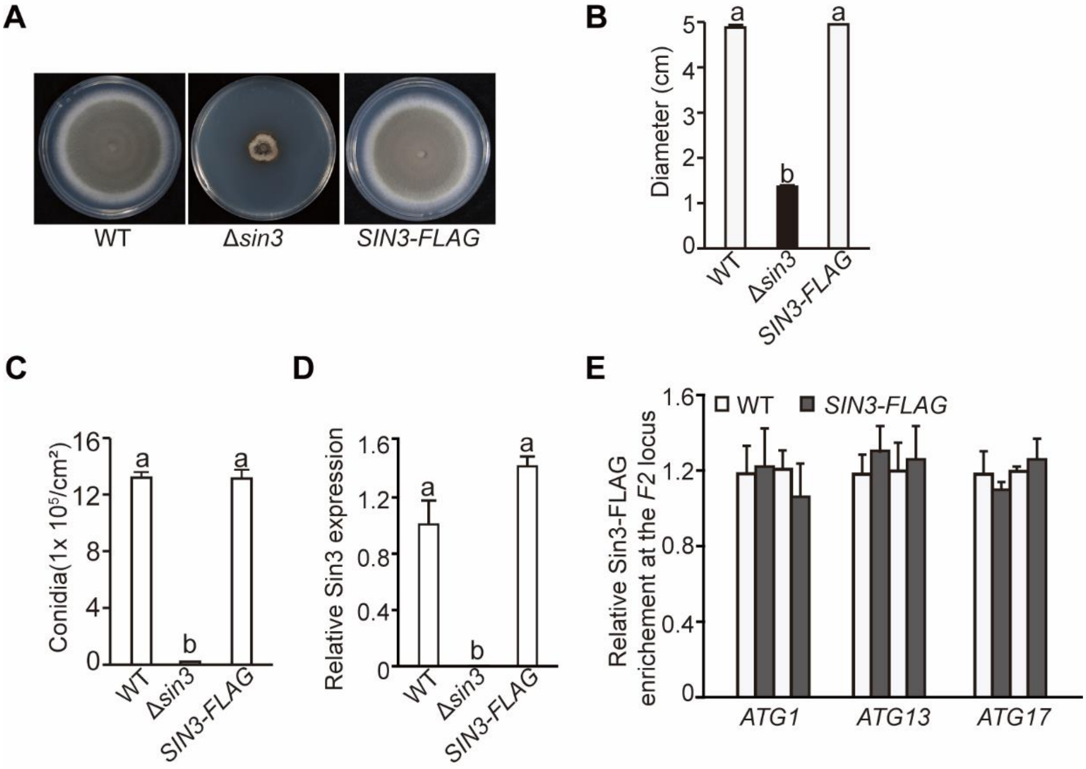

Fig. S4 Characterization of the *Sin3-FLAG* strain. (A) The colony morphology of the indicated strains grown in the CM medium for 7 d. (B-C) Statistical analysis of the colony diameter and conidiation. Different letters (a or b) indicate significant differences tested by one-way ANOVA ($P < 0.05$). Values are means ± SD from three biological replicates. (D) Analysis of *SIN3* expression of the indicated strains cultured in the CM for 2 d. (E) ChIP-qPCR analysis of relative enrichment of Sin3-FLAG in the *F2* locus of *ATG1*, *ATG13*, and *ATG17* in the WT and *Sin3-FLAG* strains. Strains were collected for ChIP experiments with FLAG antibody. Relative enrichment in the *Sin3-FLAG* strain over that in the WT strain with two independent replicates is shown. Values are means ± SD from three technical replicates.
