# Supplementary material for: Transcriptional Regulation of Autophagy-Related Genes by Sin3 Negatively Modulates Autophagy in Magnaporthe oryzae
Source: Microbiol Spectr. 2023 May 16;11(3):e00171-23. doi: 10.1128/spectrum.00171-23 (PMC10269650; doi:10.1128/spectrum.00171-23)
Supplement: Supplemental file 5 — Fig. S5. Download spectrum.00171-23-s0005.pdf, PDF file, 0.10 MB [file spectrum.00171-23-s0005.pdf]

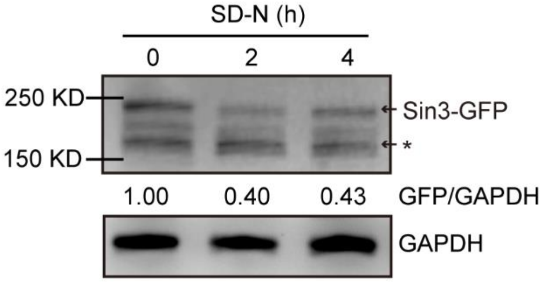


**Fig. S5** Relative abundance of Sin3-GFP detected by immunoblot analysis in the Δ*sin3*-C strain in the SD-N medium. The main band (up) of Sin3-GFP is estimated to be 190 KD. Three biological repeats were conducted with similar results. Values are means ± SD from three biological replicates. Different letters (a or b) indicate significant differences tested by one-way ANOVA ($P < 0.01$).
