# Supplementary material for: Transcriptional Regulation of Autophagy-Related Genes by Sin3 Negatively Modulates Autophagy in Magnaporthe oryzae
Source: Microbiol Spectr. 2023 May 16;11(3):e00171-23. doi: 10.1128/spectrum.00171-23 (PMC10269650; doi:10.1128/spectrum.00171-23)
Supplement: Supplemental file 6 — Fig. S6. Download spectrum.00171-23-s0006.pdf, PDF file, 0.2 MB [file spectrum.00171-23-s0006.pdf]

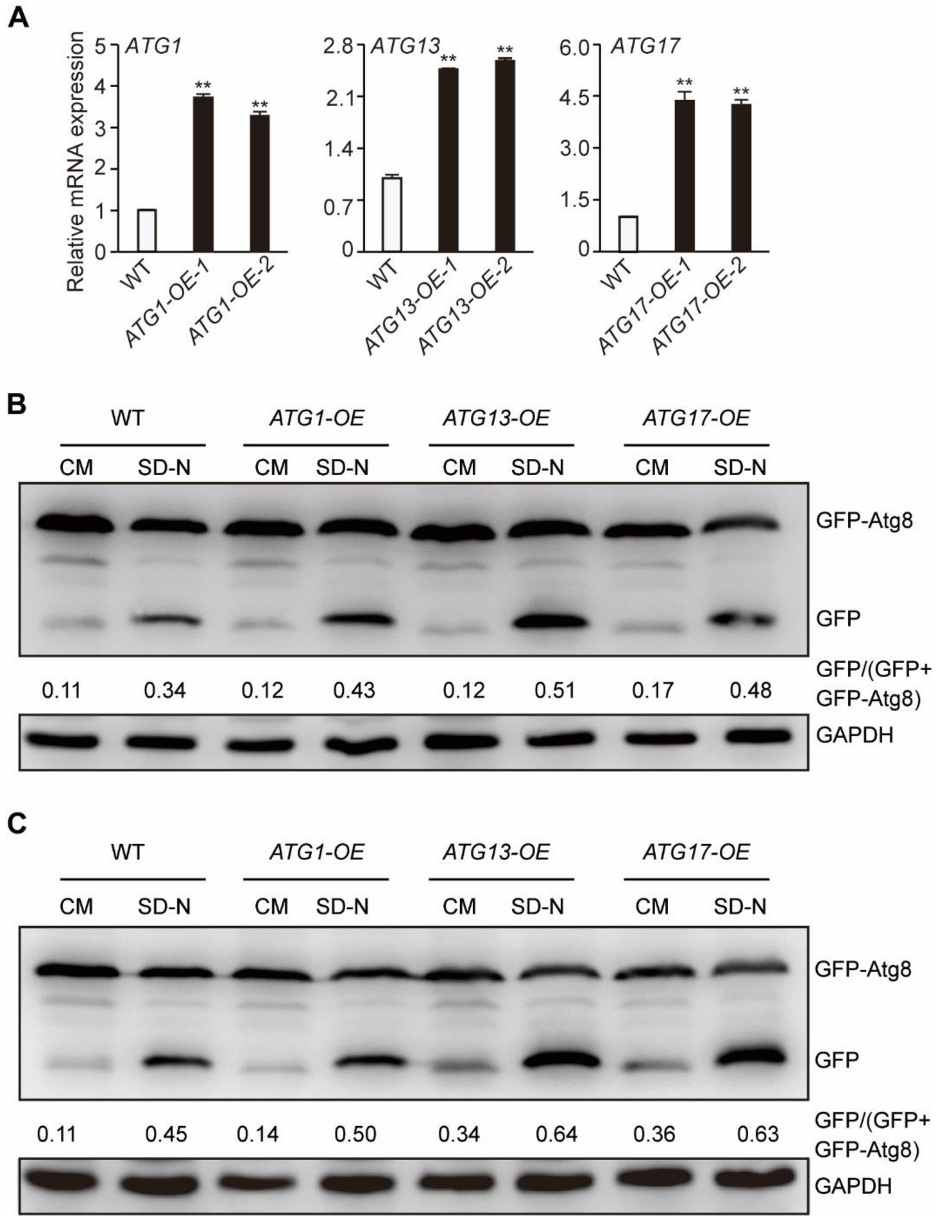

Fig. S6 Constitutive expression of *ATG1*, *ATG13*, and *ATG17* promotes autophagy in *M. oryzae*. (A) Transcriptional expression of *ATG1*, *ATG13*, and *ATG17* in the indicated strains respectively. Values are the means ± SD from three biological replicates. The asterisks indicate the significant difference (**P < 0.01). (B-C) Immunoblot analysis of GFP-Atg8 in the *GFP-ATG8, ATG1-OE/GFP-ATG8*, *ATG13-OE/GFP-ATG8*, and *ATG17-OE/GFP-ATG8* strains under CM and SD-N conditions. The degradation rates were calculated with the following formula: GFP / (GFP+GFP-Atg8). Two biological replicates were shown with similar results.
