# Supplementary material for: Transcriptional Regulation of Autophagy-Related Genes by Sin3 Negatively Modulates Autophagy in Magnaporthe oryzae
Source: Microbiol Spectr. 2023 May 16;11(3):e00171-23. doi: 10.1128/spectrum.00171-23 (PMC10269650; doi:10.1128/spectrum.00171-23)
Supplement: Supplemental file 7 — Fig. S7. Download spectrum.00171-23-s0007.pdf, PDF file, 0.2 MB [file spectrum.00171-23-s0007.pdf]

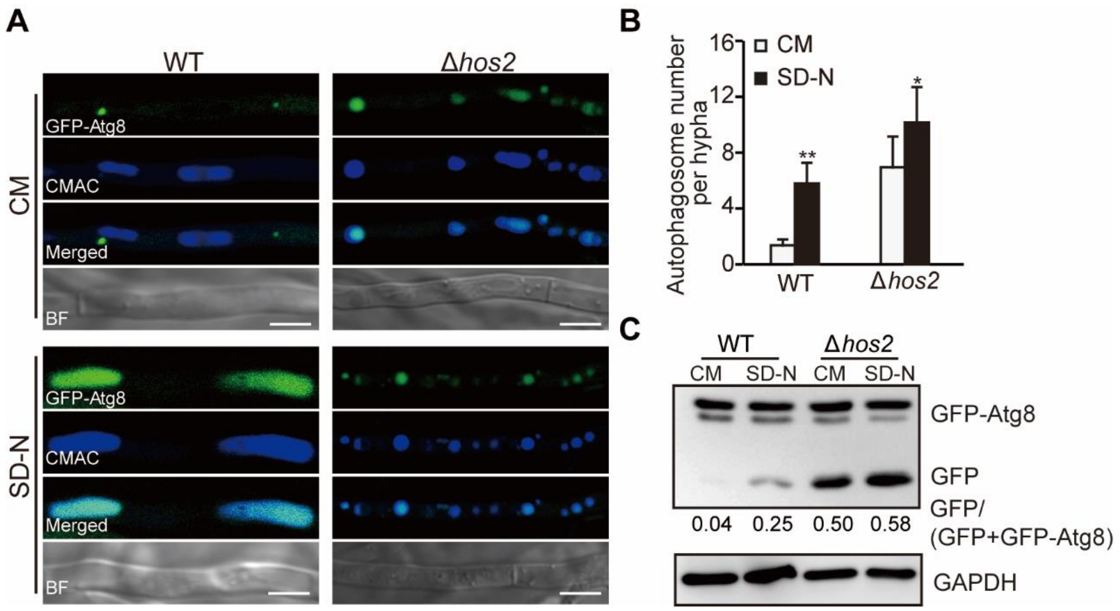

Fig. S7 Loss of *HOS2* promotes autophagy in *M. oryzae*. (A) The GFP-Atg8 localization of the WT/*GFP-ATG8* and Δ*hos2/GFP-ATG8* strains under nutrient-rich (CM) and nutrient-deficient (SD-N) conditions. Strains were cultured in the CM for 2 d, and then transferred to the SD-N for 4 h. Mycelia were stained with CMAC and images were captured by fluorescence microscopy. Bar, 20 μm. (B) The number of autophagosomes in the *GFP-ATG8* and Δ*hos2/GFP-ATG8* strains under CM and SD-N conditions. At least 25 hyphal segments were used to calculate the autophagosome. Values are means ± SD from three technical replicates. The asterisks indicate the significant difference between the WT and Δ*hos2* strains (***P* < 0.01, **P* < 0.05). (C) Immunoblot analysis of GFP-Atg8 in the *GFP-ATG8* and Δ*hos2/GFP-ATG8* strains under CM and SD-N conditions. The degradation rates were calculated with the following formula: GFP / (GFP+GFP-Atg8). Two biological replicates were performed with similar results.
