# Supplementary material for: Transcriptional Regulation of Autophagy-Related Genes by Sin3 Negatively Modulates Autophagy in Magnaporthe oryzae
Source: Microbiol Spectr. 2023 May 16;11(3):e00171-23. doi: 10.1128/spectrum.00171-23 (PMC10269650; doi:10.1128/spectrum.00171-23)
Supplement: Supplemental file 8 — Tables S1-S3. Download spectrum.00171-23-s0008.pdf, PDF file, 0.2 MB [file spectrum.00171-23-s0008.pdf]

Table S1 Strains used in this study

Strains	Description
B157	wild type strain
Δ*sin3*	deletion strain of *MGG_13498* in B157
Δ*sin3*-C	expressing $Sin3_{pro}$*-Sin3:GFP* in Δ*sin3* transformant
*Sin3-FLAG/*Δ*sin3*	expressing $RP27_{pro}$*-Sin3:FLAG* in Δ*sin3* transformant
GFP-ATG8	expressing $ATG8_{pro}$*:GFP-ATG8* in B157
Δ*sin3/GFP-ATG8*	expressing $ATG8_{pro}$*:GFP-ATG8* in Δ*sin3* transformant
Δ*hos2/GFP-ATG8*	expressing $ATG8_{pro}$*:GFP-ATG8* in Δ*hos2* transformant
ATG1-OE/GFP-ATG8	expressing $RP27_{pro}$*-ATG1* in *GFP-ATG8* transformant
ATG13-OE/GFP-ATG8	expressing $RP27_{pro}$*-ATG13* in *GFP-ATG8* transformant
ATG17-OE/GFP-ATG8	expressing $RP27_{pro}$*-ATG17* in *GFP-ATG8* transformant

Table S2 Primers used in this study

Primer name	Sequence (5’-3’)	Usage
Sin3-5F	AGTGGATTCTCAAGGCGAGTTAGGGCAG	cloning *Sin3* 5' flanking sequence for deletion
Sin3-5R	AGTGGTACCATCTCGGAGAGGGTGGTTC	
Sin3-3F	AGTCTGCAGCTTTGCAGTGACGAGCTGC	cloning *Sin3* 3' flanking sequence for deletion
Sin3-3R	AGTAAGCTTATTCTCGGTTGTGGCCCTC	
Sin3-GFP-F	TATTATGGAGAAACTCGAGAGCAAGGAAGCCCAGAAGCG	cloning *Sin3* for *Sin3-GFP*
Sin3-GFP-F	CACCATGGTACCGAGCTC TGCAGAAGACCCAGCACCG	
Sin3-Flag-F	TGACGACGATAAGCTCGAGATGGATGCAGACTCATACAGATAT	construction of *Sin3-Flag*
Sin3-Flag-R	GGAACAAAAGCTGGGTACCTTAAAACTCCATGGCTTGACCAAC	
qATG1-F	ACAGGCGGTCCAGAAGATGATTCAC	amplifying *ATG1* for RT-qPCR
qATG1-R	GCCTGATGAGGCTCTGTGGCTG	
qATG3-F	GAGGATGAAGAGGACGATGAAG	amplifying *ATG3* for RT-qPCR
qATG3-R	GGACAAGTAGAGACGAGGTG	
qATG4-F	ATTCACTGCTTACTTGCCGTCTGG	amplifying *ATG4* for RT-qPCR
qATG4-R	GGGTACTTGCCGCACTTGGC	
qATG5-F	GGGTCTTTTAGGGTCATGCAG	amplifying *ATG5* for RT-qPCR
qATG5-R	AGCAAGTCACGTAGTGTTGG	
qATG6-F	AGACATGCCGCTTGGTCTGACG	amplifying *ATG6* for RT-qPCR
qATG6-R	TATACCCAGCTTGATGCTGACGCC	
qATG7-F	TGTGGCACCTTCTGACTCAATGA	amplifying *ATG7* for RT-qPCR
qATG7-R	GCTGAAGCACGCTCGTGAGC	
qATG8-F	ATTCCCGTCATTTGCGAGAAGGTA	amplifying *ATG8* for RT-qPCR
qATG8-R	TCCTGGACGAAGATGAAGATGGC	
qATG9-F	AATGTGGAGATGGGTCAACG	amplifying *ATG9* for RT-qPCR
qATG9-R	AGGAAGGTTAGGAAAACGGC	
qATG11-F	CAGGTGTTGATAGCCCACTC	amplifying *ATG11* for RT-qPCR
qATG11-R	TGATGCAGTTCTGTGGTATCG	
qATG12-F	ACTCTCAAGGTTGGCAATGG	amplifying *ATG12* for RT-qPCR
qATG12-R	GCAGGAGTCATGGAGTAAGATAC	
qATG13-F	GCAAGACGAACAAATGGTTTCAAATA	amplifying *ATG13* for RT-qPCR
qATG13-R	CGTCATACATCTTGCCGTTATCACCG	
qATG14-F	CACCTCGGATCAACAGTCCGAA	amplifying *ATG14* for RT-qPCR
qATG14-R	ACCCTCAATGAAGAGCGAGTACATG	
qATG15-F	TGAAAGCAATGGAACGGAGAG	amplifying *ATG15* for RT-qPCR
qATG15-R	TTCATCCAAGCATCCCCAC	
qATG16-F	AGTATCTCGCGTCCATCAAAG	amplifying *ATG16* for RT-qPCR
qATG16-R	TGAGGCTCCTGATGCTTTTAG	
qATG17-F	GAGCATTGCCGAGCTCAAGGCTG	amplifying *ATG17* for RT-qPCR
qATG17-R	CGAGGTCAGCCATCGGACGG	
qATG18-F	GACTGCAACGCTAAACTTCATC	amplifying *ATG18* for RT-qPCR
qATG18-R	CGTCTTCGCTCGAGAATATCTTG	
qATG20-F	CAAGGATATGAAGAGGTTCCAGCGG	amplifying *ATG20* for RT-qPCR
qATG20-R	ACTCGTCGATCTTGTTGATCTCAACC	
qATG22-F	GGAGACGTATGTGATCTGCG	amplifying *ATG22* for RT-qPCR

qATG22-R	CTGGAGTCTTGTCGTAGCTTG	
qATG23-F	GCTGGAGAAGGTTCACGAAG	amplifying *ATG23* for RT-qPCR
qATG23-R	CTGAGGGTCCTTAATGTTCTCG	
qATG24-F	ATGATAGTTCACGGCATGGG	amplifying *ATG24* for RT-qPCR
qATG24-R	CGCATCGGGTTTTTGAAAGTC	
qATG26-F	ATGCAGCCGAACAAAGTGCCGTTCTTG	amplifying *ATG25* for RT-qPCR
qATG26-R	ATCCATCGCCGTCCTCGATCTCTGCGGC	
qATG27-F	TGACAAGGACAAGGACAAGG	amplifying *ATG27* for RT-qPCR
qATG27-R	AGAGCCAAAGATGAGGTATGC	
qATG28-F	AGAGAAAGGAGACACAACGC	amplifying *ATG28* for RT-qPCR
qATG28-R	GGGTATCTCATTATCGCTCTCC	
qATG29-F	AGCTCTGTGGAACATTCTGTC	amplifying *ATG29* for RT-qPCR
qATG29-R	ATGGCGTTCTGTAAGGTACG	
qRS2-F	TCGTTCAGCCCGTCAGCCAA	amplifying *RS2* for RT-qPCR
qRS2-R	CTCCTGCTCCTCACCCTCACCC	
qRS3-F	GGTTGCCTCGCCCGCTG	amplifying *RS3* for RT-qPCR
qRS3-R	CGCTTGCCGTCCCTGAGG	
qeIF4G-F	GGAGTCGGGTGCGTCGTCA	amplifying *eIF4G* for RT-qPCR
qeIF4G	TTATTTCTTGTCTTTATCGCCAGTGTCAG	
qβ-TUBULIN-F	CTGCCATCTTCCGTGGAAAGG	amplifying *β-TUBULIN* for RT-qPCR
qβ-TUBULIN-R	GACGAAGTACGACGAGTTCTTG	
Atg1-OE-F	TGACGACGATAAGCTCGAGATGGCGGACCGATCAGCACG	construction of *ATG1-OE*
Atg1-OE-R	GGAACAAAAGCTGGGTACCTCAAGAGCTGTATGAAGGGACACT	
Atg13-OE-F	TGACGACGATAAGCTCGAGATGCATCAACAGTCCCGCTATCCT	construction of *ATG13-OE*
Atg13-OE-R	GGAACAAAAGCTGGGTACCTCACCAGCCTCGCCTTGTGAT	
Atg17-OE-F	TGACGACGATAAGCTCGAGATGCCGTCTTCAAGTTCCGCC	construction of *ATG17-OE*
Atg17-OE-R	GGAACAAAAGCTGGGTACCTCAACGTCCATGTACCCTCTCCCT	
ChIP-ATG1-F	TCACGACTTGACGATAACTTTCACATT	amplifying *ATG1* for ChIP-qPCR
ChIP-ATG1-R	CGTCCTCAGCACCAAGTTGCG	
ChIP-ATG13-F	GTTCTGACACCTCCGGCTATCAGC	amplifying *ATG13* for ChIP-qPCR
ChIP-ATG13-R	GACAGAAGCAGAAGCAATCCGG	
ChIP-ATG17-F	TCATCGACACTTGCGAATGTGTTG	amplifying *ATG17* for ChIP-qPCR
ChIP-ATG17-R	TTTGAAGACGATGACGATGCGG	
ChIP-ATG1-3utr-F	TCACGACTTGACGATAACTTTCACATT	amplifying *ATG1-3'UTR* for ChIP-qPCR
ChIP-ATG1-3utr-R	CGTCCTCAGCACCAAGTTGCG	
ChIP-ATG13-3utr-F	CCAGGACAGAAGACCCTGTGTAGCC	amplifying *ATG13-3'UTR* for ChIP-qPCR
ChIP-ATG13-3utr-R	CCAAAGTAGCAATGGCGAAACTCC	
ChIP-ATG17-3utr-F	TGATGTTCTTTGTCGACGAGGGC	amplifying *ATG17-3'UTR* for ChIP-qPCR
ChIP-ATG17-3utr-R	ATATGAAGCAAGACAAACCTTGAGC	

Table S3 Probe sequences in EMSA

EMSA-ATG1-Biotin-F：
ACTGACACGAATGCAGCCTTTTGACAGAAGTCAACCTTCCTAGGCCACAAAACTCTTA
TTACTCTGCTTGCTCGGTCCTAGATCTGGACGTACAGTAGGT
EMSA-ATG1-R：
ACCTACTGTACGTCCAGATCTAGGACCGAGCAAGCAGAGTAATAAGAGTTTTGTGGC
CTAGGAAGGTTGACTTCTGTCAAAAGGCTGCATTCGTGTCAGT

EMSA-ATG13-Biotin-F:
GATGATGGCTCCGGATAAGAGGGCGCTGCAGATGCATCCATCTATCGGTAGTCTGCTT
GGGTTGGAAGGCTTATTTTGTTGTTCTTTTTTCTTGGTACTG
EMSA-ATG13-Biotin-R:
CAGTACCAAGAAAAAAGAACAACAAAATAAGCCTTCCAACCCAAGCAGACTACCGA
TAGATGGATGCATCTGCAGCGCCCTCTTATCCGGAGCCATCATC

EMSA-ATG17-Biotin-F:
TGGCAGAGGGTTTCTGCGCGTTGGACCGGGGCGCACCTGAAGACTGCCCATCGTTCA
GTTGGCGCTGCCGGTTGTAGGTTTTGGTTAGGTCGCCGGTTGT
EMSA-ATG17-Biotin-R:
ACAACCGGCGACCTAACCAAAACCTACAACCGGCAGCGCCAACTGAACGATGGGCA
GTCTTCAGGTGCGCCCCGGTCCAACGCGCAGAAACCCTCTGCCA
